# Planning policies to restrict fast food and inequalities in child weight in England: a quasi‐experimental analysis

**DOI:** 10.1002/oby.24127

**Published:** 2024-10-23

**Authors:** Huasheng Xiang, Louis Goffe, Viviana Albani, Nasima Akhter, Amelia A. Lake, Heather Brown

**Affiliations:** ^1^ Division of Health Research, Lancaster University Lancaster UK; ^2^ Health Determinants Research Collaborative (HDRC) Gateshead, Fuse Centre for Translational Research in Public Health Gateshead UK; ^3^ Population Health Sciences Institute, Fuse Centre for Translational Research in Public Health, Newcastle University Newcastle Upon Tyne UK; ^4^ Fuse Centre for Translational Research in Public Health Teesside University Middlesbrough UK

## Abstract

**Objective:**

England has one of the highest childhood obesity rates in Europe. To promote a healthier food environment in 2015, Gateshead Council in North East England introduced planning guidelines effectively banning any new fast‐food outlets. Our aim was to investigate whether this policy led to any reductions in childhood overweight and obesity prevalence and the inequalities in these outcomes.

**Methods:**

We used data from the National Child Measurement Programme, the Food Standards Agency Food Hygiene Rating Scheme data, and the Office of National Statistics between 2012 and 2020. We estimated a difference‐in‐differences model employing propensity score matching to identify a control group.

**Results:**

We found no significant change in population‐level childhood overweight and obesity in Gateshead compared with control areas. In subgroup analysis by area‐level deprivation, we found that the quintile of deprivation with the highest proportion of fast‐food outlets had a statistically significant reduction of 4.8% in the prevalence of childhood overweight and obesity compared with control areas.

**Conclusions:**

Restricting fast‐food outlets in areas with a high concentration of such outlets as part of a package of policies to reduce childhood obesity may help to reduce prevalence and inequalities in childhood overweight and obesity.

**Study Importance**

**What is already known?**

- Using planning policy to restrict food outlets reduces the number of fast‐food outlets.
- Public health impact of these policies is not known.

**What does this study add?**

- In local areas with the highest concentration of fast‐food outlets, restricting new outlets significantly reduces the prevalence of childhood overweight and obesity compared with control areas.
- There is no impact of the policy on childhood overweight and obesity at the population level.

**How might these results change the direction of research?**

- Planning policy may be an effective tool to reduce childhood overweight and obesity rates in areas with a high concentration of outlets. This is the first study from the UK, to our knowledge, to provide evidence on the public health impact of planning policy to shape the food environment.

## INTRODUCTION

Childhood obesity rates in the UK are some of the highest in Europe [1]. In 2006 and 2007, 31.7% of children in year 6 (ages 10 to 11 years) were living with overweight and obesity (OWOB), which rose to 35.2% in 2019 and 2020 and further increased to 40.9% in 2020 and 2021 [2]. This rise has been partially exacerbated by the COVID‐19 pandemic [3].

There has been robust evidence showing that childhood obesity can have adverse impacts on health in the short term (childhood) and long term (adulthood). Obesity in childhood is associated with increased risk for anxiety and depression, low self‐esteem, lower reported quality of life, increased risk of bullying and facing stigma, and increased risk of obesity in adulthood [4, 5, 6]. Childhood obesity is strongly associated with increased risk of type 2 diabetes, cardiovascular diseases, and mental disorders in adulthood [7, 8, 9, 10]. The estimated costs to the National Health Service (NHS) for treating OWOB‐related diseases was £6.1 million ($6.6 million) in 2015 and is forecasted to reach £9.7 billion ($10.4 billion) by 2050 [11].

The causes of childhood obesity are complex and multifaceted. However, environmental factors play an important role in the prevalence of childhood obesity [12, 13]. There has been evidence showing that the out‐of‐home food environment has impacts on childhood energy intakes [14, 15, 16, 17]. In particular, fast‐food consumption and the location of fast‐food outlets are strongly associated with a higher energy intake and a higher prevalence of childhood obesity [17, 18, 19, 20]. The relationship with obesity is also strongly sociodemographically and socioeconomically patterned, with the highest prevalence of OWOB found in the most economically deprived communities [21, 22, 23, 24].

The density of fast‐food outlets is the number of fast‐food outlets per 100,000 residents [25], and it has been rising across England. Data from the Food Standards Agency show that the average density of fast‐food outlets increased from 142 to 170 per 100,000 residents between 2019 to the end of 2021. Areas of higher deprivation have five times as many fast‐food outlets compared with more affluent areas [26]. This may be a contributing factor to inequalities in childhood weight.

Since the enactment of the Health and Social Care Act 2012, local authorities (i.e., local government) in England have had a statutory duty regarding improvement of population health [27]. Because of the clear and consistent evidence base demonstrating a relationship between childhood obesity and the food environment [15, 17], national public health guidance was developed to encourage and support local authorities to use the planning system to create environments that are supportive of promoting a healthy weight [28]. Approximately 50% of local authorities have employed planning guidelines restricting planning permission for new fast‐food outlets to promote a healthier food environment [29]. There are three different types of planning guidelines used by local authorities, outlined in the online Supporting Information.

In England, for planning purposes, fast‐food outlets are defined as premises that sell hot food for consumption off the premises (Town and Country Planning [Use Classes] Order 1987 [as amended]). However, the data used to monitor the food environment by the UK public health agency (Office for Health Improvement and DIsparities) are based on the Food Standards Agency data collected by environmental health officers. In our analysis, we use this definition to classify fast‐food outlets. Fast‐food outlets in our study include businesses that, for planning terms, would be considered planning class E, such as sushi bars and sandwich shops; however, in terms of public health, these outlets were considered fast food.

In 2015, Gateshead implemented all three types of planning guidance (online Supporting Information). Gateshead is in the top 15% of the most deprived local authorities in England [30], and it is located in North East England. In 2014, 36.7% of year 6 children (ages 10 to 11 years) were living with OWOB in Gateshead compared with 36.1% in the North East and 33.5% in England [31]. The Gateshead policy is equivalent to a blanket‐ban on obtaining planning permission for change of use or building of a new premise to be designated as a fast‐food outlet. The ambition of the policy is to reduce the year 6 (10– to 11‐year‐old children) obesity rate from 23% in 2015 to less than 10% by 2025 [32]. Research has found that the planning guidance led to a statistically significant reduction in the density and proportion of fast‐food outlets in Gateshead compared with other neighboring local authorities that did not have similar planning policy in place [33].

The aim of this paper is to explore whether a reduction in fast‐food outlets is associated with a change in childhood OWOB and inequalities in childhood OWOB. We know that the food environment has an indirect effect on body weight by influencing what food is available and what is subsequently consumed. We do not know how long it takes for the changes in the food environment to filter down to observed changes in weight. Therefore, our first objective is to explore whether, at the population level, there is a significant change in childhood OWOB within the first 5 years of a change in planning guidance. We know that, on average, more deprived areas have a higher concentration of fast‐food outlets and that the density and proportion of fast‐food outlets have decreased in Gateshead as a result of the change in planning guidance [33]. Our second objective is to explore whether the policy is more effective in areas of higher deprivation that previously had a higher concentration of outlets, leading to a reduction in inequalities in childhood OWOB.

There is currently limited evidence on the effectiveness of planning policy on health outcomes. Understanding how and for whom planning policy works is essential so that local government can use planning policy as a cost‐effective mechanism to improve population health and reduce health inequalities.

## METHODS

### Data sources

All datasets used in this study are publicly available. The pretreatment period is 2011 through 2014, and the posttreatment period is 2015 through 2019. We exclude data from the COVID‐19 period because data on child weight were not collected for all children in 2020 and 2021 [2]. Data on children's weight came from the National Child Measurement Programme (NCMP) from 2011 to 2020. NCMP is a statutory program delivered annually by NHS Primary Care Trusts before 2013 and by local authorities after 2013 that collects data on the height and weight of all schoolchildren in reception (ages 4–5 years) and year 6 (ages 10–11 years) across England [34]. Children are classified as having overweight if their body mass index (BMI) is at or above the 85th percentile (or 95th percentile for obesity) of the British 1990 growth reference according to age and sex [35].

Data on food outlets were from the Food Standards Agency Food Hygiene Rating Scheme (FSA FHRS) between 2012 and 2020 [36]. We did not use data after 2020 because of the COVID‐19 pandemic and the resulting temporary changes brought into planning guidance and changes to how food businesses could operate [37, 38]. The FSA FHRS records information (including business name, type of food outlet, location, and hygiene rating) on all premises that serve hot food in the UK, and it is updated regularly (between 4 and 8 weeks). All premises that serve hot food must register with the local authority at least 28 days before opening [39]. Premises will then be inspected by an environmental health officer from the local authority and given a food hygiene rating. Subsequent inspections will occur between every 6 months to every 2 years depending upon the potential risk to public health from the food premises [36]. There is evidence that the FSA FHRS dataset has a broad coverage of food outlets and a high spatial accuracy of the food environment in North East England [40].

We cross‐checked our data based on the planning guidance to ensure that we were not missing any outlets [32]. Our data are a conservative estimate of the number of fast‐food outlets because the FSA/environmental health definition is broader than the planner's definition.

We also used data on population size between 2012 and 2020 [41] and Index of Multiple Deprivation (IMD) 2019 [42]. IMD is a composite measure of seven distinct domains of deprivation that include the following: 1) income; 2) employment; 3) health and disability; 4) education, skills, and training; 5) crime; 6) barriers to housing and services; and 7) living environment.

### Geography

We undertook all analyses at the middle layer super output area (MSOA) level because the NCMP data are not publicly available at a smaller geography. An MSOA is a geographical area with an average population of 7200 people [43].

### Outcome variable: prevalence of year 6 OWOB

The main outcome of interest is the prevalence of OWOB for children in year 6 (ages 10–11 years). The prevalence of OWOB was the ratio of the number of children living with OWOB to the total number of children who had height and weight data. In our sample, NCMP collected an average of 232 children's weight in each MSOA and year (a total of 425,715 children over the sample years). Table A1 in online Supporting Information presents the number of children in each MSOA in Gateshead over the study period.

### Density of fast‐food outlets

We calculated the density of fast‐food outlets by MSOA and year between 2012 and 2020. It is defined as the number of fast‐food outlets per 100,000 residents. A higher density of fast‐food outlets indicates that the year 6 children have a higher exposure to unhealthy food. This measure has been used in previous studies [25, 33]. To count the number of fast‐food outlets within each MSOA, we extracted the postcode and location information of all fast‐food outlets from the FSA FHRS dataset [36]. We have data on 13,074 food outlets in the control and treatment groups over the study period. A food outlet may have multiple observations over the study period. The population in each MSOA was estimated from data by the Office for National Statistics in 2021 [41].

### Area‐level deprivation

To measure the relative deprivation for each MSOA, we used a population‐weighted IMD score following the method described in the English Indices of Deprivation 2019 research report [44]. A higher IMD score indicates a higher level of deprivation. Then, we ranked the IMD scores to identify the IMD quintiles. The first IMD quintile is the most deprived MSOAs, and the fifth IMD quintile is the least deprived MSOAs in Gateshead.

### Identification of control groups

MSOAs in Gateshead are the areas that underwent the planning changes (treatment group). To identify an appropriate group of MSOAs for comparison (control units), the selection of control groups is restricted to the MSOAs located in North East England belonging to local authorities that had not adopted any of the three types of planning guidance over the study period. There are five local authorities that met the criteria: 1) Stockton on Tees; 2) Durham; 3) Northumberland; 4) Darlington; and 5) Hartlepool. Durham and Hartlepool were within the 20% most deprived local authorities in England. The other three local authorities were within the 40% most deprived local authorities in England [44].

**FIGURE 1:**
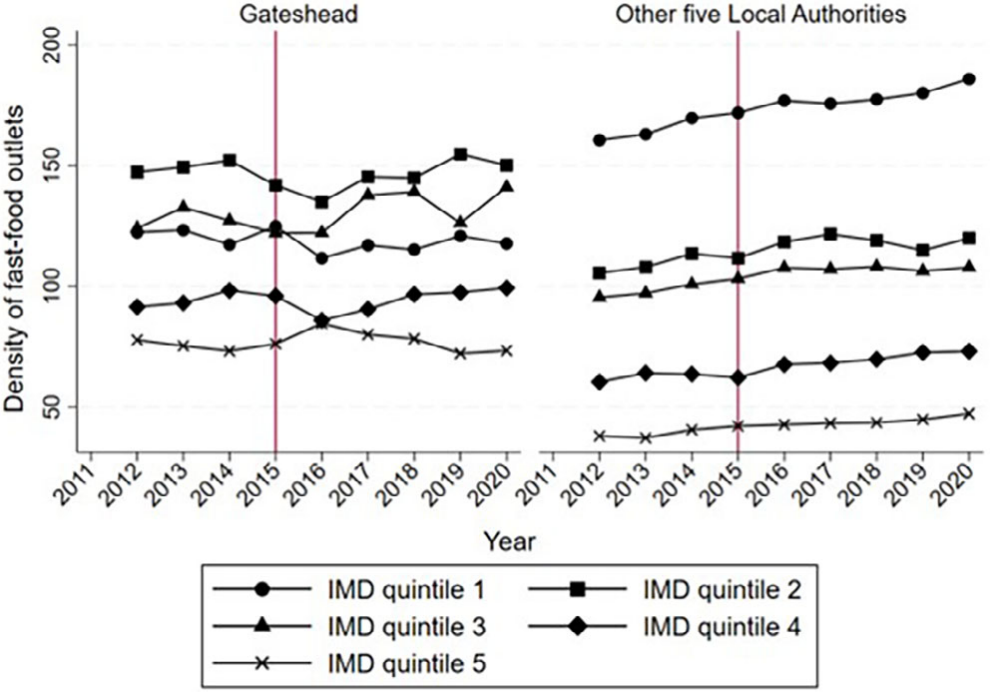
Density of fast‐food outlets by Index of Multiple Deprivation (IMD) quintile. IMD quintile 1 refers to the 25% most deprived middle layer super output areas (MSOAs) in the control and treatment groups. IMD quintile 5 refers to the 25% least deprived MSOAs in the control and treatment groups. [Color figure can be viewed at wileyonlinelibrary.com]

The decision to use planning policy restricting new fast‐food outlets was not a coincidence given that Gateshead had a higher density of fast‐food outlets and a higher level of deprivation compared with the other five local authorities in the North East, as shown in Table 1. Furthermore, there is significant heterogeneity in the distribution of fast‐food outlets between the MSOAs in Gateshead and the other five local authorities. In Figure 1, we identified the density of fast‐food outlets by IMD quintile. In both groups, MSOAs with a higher level of deprivation tend to have a higher density of fast‐food outlets. However, the first IMD quintile of MSOAs in Gateshead had a lower density of fast‐food outlets than the second and third IMD quintiles. The MSOAs in Gateshead also had a relatively higher variation in the density of fast‐food outlets over time. These dynamics make it difficult to identify an appropriate control group.

**TABLE 1:** Characteristics of MSOAs in the treatment and control groups before and after PSM.

	Treatment: full sample		Control: full sample		Control: matched		*t* tests	
	Mean	SD	Mean	SD	Mean	SD	Diff.	*p* value
	1	2	3	4	5	6	7	8
IMD 2019 scores	28.25	12.07	26.29	13.60	28.67	13.34	0.42	0.90
Density of fast‐food outlets	113.82	77.13	95.39	94.45	107.86	77.14	−5.97	0.78
Number of MSOAs	27		156		27			
Number of fast‐food outlets	2109		10,965		2263			
Number of children	55,440		370,275		22,770			

*Note*: Results from the *t* tests, columns 7 and 8, show the differences between the matched control (i.e., column 5) and treatment MSOAs (i.e., column 1). Number of fast‐food outlets reports the total number of fast‐food outlets observed over the study period. A fast‐food outlet may be repeatedly observed in different years. Number of children shows the total number of children observed over the whole study period.

Abbreviations: Diff., difference; IMD, Index of Multiple Deprivation; MSOA, middle layer super output area; PSM, propensity score matching.

To overcome the heterogeneous fast‐food outlet distribution, we employed a propensity score matching (PSM) approach. A one‐to‐one matching without replacement was performed. Specifically, using the preintervention data and a logit regression model, we employed the average density of fast‐food outlets and IMD scores as predictors to estimate the propensity scores for MSOAs. There are 27 MSOAs in Gateshead; therefore, 27 MSOAs from the other five local authorities with the nearest propensity scores were identified as the control groups.

Table 1 compares the characteristics of MSOAs in the treatment and control groups before and after the matching. As shown, before the matching, the control groups have a lower IMD score with a higher standard deviation (SD) and a lower density of fast‐food outlets with a higher SD compared with the treatment groups. After the matching, the differences between the control and treatment groups become smaller, as shown in columns 5 and 6. Results from *t* tests show that there are no statistically significant differences in IMD scores and density of fast‐food outlets between the matched control and treatment MSOAs. This suggests that we might have identified an appropriate group of MSOAs as the control groups.

**FIGURE 2:**
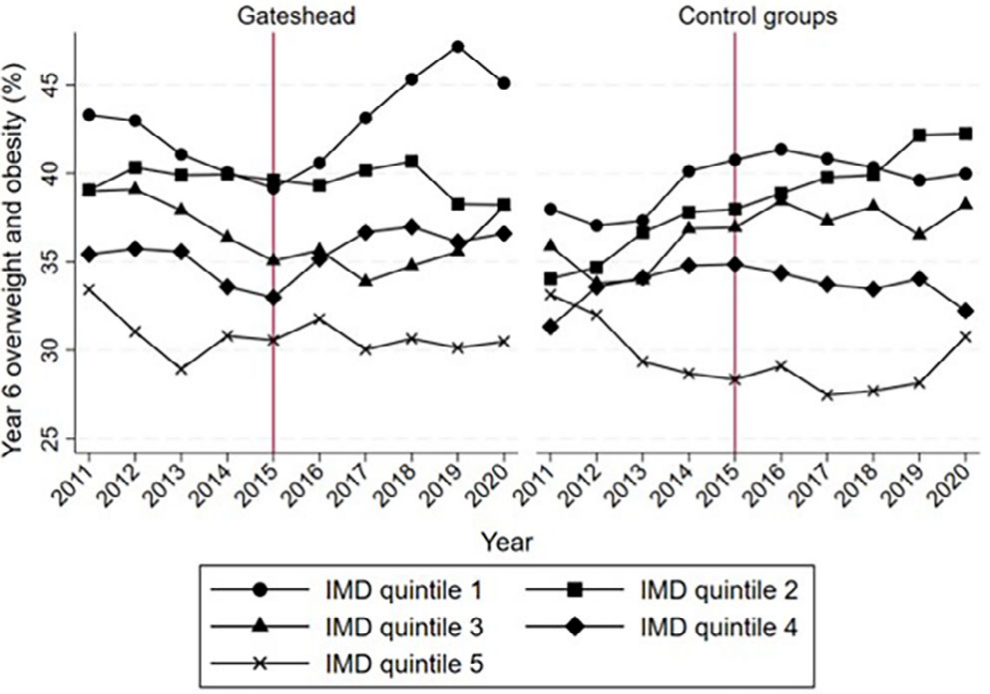
Prevalence of year 6 overweight and obesity (OWOB) by Index of Multiple Deprivation (IMD) quintile over time. IMD quintile 1 refers to the 25% most deprived middle layer super output areas (MSOAs) in Gateshead. IMD quintile 5 refers to the 25% least deprived MSOAs in Gateshead. [Color figure can be viewed at wileyonlinelibrary.com]

Figure 2 displays the prevalence of year 6 OWOB by IMD quintile over time. The most deprived MSOAs (i.e., IMD quintile 1) tend to have the highest prevalence of year 6 OWOB, and the least deprived MSOAs (i.e., IMD quintile 5) tend to have the lowest prevalence of year 6 OWOB in all years.

The inequality in childhood OWOB in Gateshead is relatively constant for IMD quintiles 2 through 5 but increases sharply for those in IMD quintile 1 (most deprived) from 2016. The most deprived MSOAs in Gateshead, on average, have a higher turnover in housing [30]. This suggests that the increase in childhood OWOB that we observe may be because of a change in the sample composition. However, because we use aggregate data, we cannot explore this in the data. For the control groups, inequality between IMD quintiles is increasing up until 2019, when it stabilizes or starts to decrease.

### Econometric analysis

This study employs a difference‐in‐differences (DID) model to quantitively examine the changes in the prevalence of OWOB in children in year 6 in Gateshead compared with other, similar local authorities by the relative deprivation quintile. The Gateshead policy was adopted in 2015. Therefore, the pretreatment period is 2012 through 2015, and the posttreatment period is 2015 through 2020. We started with modeling the overall relationship between the policy intervention and the prevalence of childhood OWOB using the matched sample from the PSM model. Equation (1) shows the model. All analyses were conducted using Stata version 17 (StataCorp LLC).

(1) $${\text{OWOB}}_{\mathrm{it}}=\alpha +\delta_{1}{\text{Treat}}_{i}+\delta_{2}{\text{Post}}_{t}+\beta {\text{Treat}}_{i}*{\text{Post}}_{t}+\gamma {\mathrm{Dep}}_{i}+\varepsilon_{\mathrm{it}}$$

In Equation 1, the subscripts *i* and *t* indicate an MSOA and year, respectively; OWOB_*it*_ is the prevalence of year 6 OWOB in MSOA *i* in year *t*; Treat_*i*_ is a dummy variable that is set to 1 if MSOA *i* is within Gateshead and 0 otherwise; Post_*t*_ is a dummy variable that is set to 1 for the postintervention years and 0 for the preintervention years; Treat*Post is the impact of the intervention on childhood OWOB rates in MSOAs in Gateshead; Dep_*i*_ indicates the IMD quintile for MSOA *i*; *ε*_it_ is the error term; and α is the constant term. *β*, *δ*_1_, *δ*_2_, and *γ* are the parameters of coefficients to be estimated. We expect *β* to be negative if the planning policy has had positive impacts on reducing the prevalence of childhood OWOB.

Because fast‐food outlets tend to cluster in more deprived areas, the impact of the planning policy on year 6 OWOB is likely to partially depend on area‐level deprivation. To observe the heterogeneous effects of the policy, we also estimated the DID model by the IMD quintile.

### Sensitivity analysis

We perform a range of different tests on the robustness of our results, checking the underlying assumptions of the model. This sensitivity analysis is described in greater detail in online Supporting Information.

## RESULTS

### Summary statistics

Table 2 reports the prevalence of year 6 OWOB, the number and density of fast‐food outlets, and the number of children's weights collected over the 27 MSOAs in Gateshead and the 156 in the five control local authorities by year. Gateshead's MSOAs had a higher prevalence of year 6 OWOB in all years compared with MSOAs in the other five local authorities. We observed a decreasing trend in OWOB in Gateshead pre intervention, from 38% in 2011 to 35.5% in 2015, which then reversed to 37.7% in 2020. In the control MSOAs, the prevalence of year 6 OWOB was increasing throughout the sample years, from 34.4% in 2011% to 36.4% in 2020. However, in general, the differences between these two groups became smaller over time. The second and third rows in each panel show the number and density of fast‐food outlets. Gateshead MSOAs had a higher density of fast‐food outlets compared with the five control local authorities in all years.

**TABLE 2:** Characteristics of MSOAs in Gateshead and five control local authorities in North East England.

Gateshead MSOAs	2011	2012	2013	2014	2015	2016	2017	2018	2019	2020
	1	2	3	4	5	6	7	8	9	10
% of year 6 OWOB	38.0	37.9	36.8	36.2	35.5	36.6	36.9	37.8	37.4	37.7
Total number of fast‐food outlets	‐	230	234	233	231	222	237	239	240	243
Density of fast‐food outlets	‐	113.1	115.2	114.4	112.6	108.0	114.4	115.2	115.1	116.9
Total number of children	5465	5335	5385	5385	5400	5495	5605	5710	5760	5900
Number of MSOAs	27	27	27	27	27	27	27	27	27	27

Other MSOAs	2011	2012	2013	2014	2015	2016	2017	2018	2019	2020
	1	2	3	4	5	6	7	8	9	10
% of year 6 OWOB	34.4	35.0	35.3	35.3	35.1	35.5	36.0	36.4	36.4	36.4
Total number of fast‐food outlets	‐	1109	1130	1178	1190	1249	1260	1269	1273	1307
Density of fast‐food outlets	‐	91.9	93.8	97.6	98.2	102.6	103.1	103.5	103.7	106.8
Total number of children	37,015	36,200	35,125	35,220	36,185	37,175	38,095	38,710	39,590	36,960
Number of MSOAs	156	156	156	156	156	156	156	156	156	156

*Note*: This table shows the prevalence of year 6 OWOB, number of fast‐food outlets, density of fast‐food outlets, and number of children across the treatment MSOAs and the control MSOAs over the study period.

Abbreviations: MSOA, middle layer super output area; OWOB, overweight and obesity.

### Population‐level results

Table 3 reports the estimates of the impact of planning guidance on year 6 OWOB on the matched MSOAs. Columns 1 and 2 include different sets of covariates. As shown in Table 3, the relationship between the planning guidance and the prevalence of year 6 OWOB is not statistically significant in any model (first two rows). In the bottom half of Table 3, we can see that there is a strong and significant gradient in the prevalence of year 6 OWOB by area‐level deprivation. Compared with the most deprived quintile of MSOAs (IMD quintile 1), the prevalence of year 6 OWOB in the IMD quintiles 2, 3, 4, and 5 is 2.2%, 4.6%, 6.6%, and 11.0% lower, respectively. The *R* ^2^ increased from 0.025 to 0.440, suggesting that the IMD may explain more than 40% of the prevalence of year 6 OWOB.

**TABLE 3:** The impact of planning policy on year 6 OWOB estimated using a DID equation.

	Base model (1)	Includes IMD (2)
Gateshead (treat)	1.976*** (0.616)	1.976*** (0.488)
Post (implementation of SPD)	1.365* (0.712)	1.365** (0.571)
Treat*Post	−0.961 (0.981)	−0.961 (0.746)
IMD quintile		
Quintile 2		−2.181*** (0.555)
Quintile 3		−4.587*** (0.593)
Quintile 4		−6.595*** (0.594)
Quintile 5		−11.031*** (0.686)
*N* (number of MSOAs × years)	540	540
*R* ^2^	0.025	0.440

*Note*: IMD quintile 1 is an indicator of the most deprived MSOAs and is omitted in this table. IMD quintile 5 is an indicator of the least deprived MSOAs. Constants are included but not reported. Robust standard errors are shown in parentheses. *N* is the number of MSOAs × years.

Abbreviations: DID, difference‐in‐differences; IMD, Index of Multiple Deprivation; MSOA, middle layer super output area; OWOB, overweight and obesity; SPD, Supplementary Planning Document.

* *p* < 0.10.

** *p* < 0.05.

*** *p* < 0.01.

### Heterogeneous effects of the planning policy by area‐level deprivation

Because of the significant association between area‐level deprivation and childhood OWOB in Table 3, in Table 4, we estimated the impact of the policy by area‐level deprivation. Examined by the most deprived quintile of MSOAs, we observed that the prevalence of year 6 OWOB had a statistically significant reduction of 4.8% (*p* < 0.01) in the second IMD quintile and 4.1% (*p* < 0.01) in the third IMD quintile in Gateshead following the adoption of the planning policy compared with the control groups. In other IMD quintiles, there were no statistically significant changes in the prevalence of year 6 OWOB in Gateshead compared with the control groups after the adoption of the planning guidance.

**TABLE 4:** The impact of planning policy on childhood OWOB by IMD quintile estimated by a DID equation.

	Q1	Q2	Q3	Q4	Q5
	1	2	3	4	5
Gateshead (treat)	2.669** (1.036)	3.537*** (0.991)	1.986* (1.156)	0.930 (1.031)	0.652 (1.211)
Post (implementation of SPD)	1.778 (1.082)	4.357*** (0.892)	2.225* (1.207)	−0.165 (1.314)	−1.661 (1.688)
Treat*Post	1.168 (1.715)	−4.789*** (1.289)	−4.106*** (1.527)	1.811 (1.580)	1.321 (2.075)
*N* (number of MSOAs × years)	100	120	100	120	100
*R* ^2^	0.189	0.184	0.071	0.061	0.030

*Note*: Q1 through Q5 refer to the IMD quintile 1 through 5, respectively. Constants are included but not reported. Robust standard errors are shown in parentheses. *N* is the number of MSOAs × years.

Abbreviations: DID, difference‐in‐differences; IMD, Index of Multiple Deprivation; MSOA, middle layer super output area; OWOB, overweight and obesity; SPD, Supplementary Planning Document.

* *p* < 0.10.

** *p* < 0.05.

*** *p* < 0.01.

### Sensitivity analysis

The results for the sensitivity analysis are presented in online Supporting Information. The results suggest heterogeneity in the robustness of the results by IMD quintile. The results for IMD quintile 2 are robust across all sensitivity analyses, providing confidence that the policy was associated with the reduction of childhood OWOB for children in these areas compared with the control group.

## DISCUSSION

In this study, we provided empirical evidence on the effectiveness of local planning policy on reducing the prevalence of childhood OWOB at ages 10 to 11 years (year 6) within a 4‐year postimplementation period. We know that there is a higher concentration of fast‐food outlets in areas of high deprivation [26]. There has been evidence to suggest that children from more deprived areas are more likely to have OWOB [2, 21, 24]. Across all model specifications, we found that, for children living in the second most deprived quintile (which had the highest concentration of fast‐food outlets pre intervention), there was a decrease in the prevalence of OWOB compared with control groups. Results from other quintiles of MSOAs are less robust to alternative specifications.

At the population level, we found no significant impact of the policy on childhood OWOB in Gateshead compared with the control areas in this relatively short time period. This may be because the policy had no effect or the effect of the policy may happen in the longer term; therefore, we did not observe a change within 4 years of post‐policy data that we used in our analysis. Evidence from Gateshead showed a 10% reduction in the density and proportion of fast‐food outlets within 4 years of the policy intervention [33].

The food environment is changing, with a growing presence of online food delivery available. Future research needs to consider whether and how the online food environment may impact the exposure to unhealthy food and whether existing guidance and legislation needs to be changed to reflect this changing food environment.

### Policy implications

Our finding of a significant association between a decrease in childhood OWOB and a reduction in the density of fast food in a more deprived quintile with a higher pre‐policy density of fast‐food outlets is important to highlight how planning policy may contribute to reducing inequalities in childhood OWOB. This means that planning policy may be an effective mechanism to reduce inequalities in childhood OWOB, a policy goal that has not been achieved to date despite the long history of government policy to increase the prevalence of healthy‐weight children [45]. Our results provide evidence to support an “upstream” or structural approach to improving health outcomes rather than a reliance on individuals as drivers of behavior change.

Planning policy has direct impacts on the food environment that then have indirect impacts on weight. A priori, it is difficult to know how long it may take for this change in the food environment to impact energy intake from food, resulting in changes to weight.

### Strengths and limitations

The present study offers several notable strengths, such as the fact that we employed a quasi‐experimental method with sensitivity analysis.

Despite these strengths, several limitations should be acknowledged. We were unable to identify the time frame required for changes in the food environment to translate into observed changes in childhood OWOB. Although efforts were made to control MSOA characteristics, we were unable to incorporate some time‐varying variables such as changing sample composition. The outcome measure contains the weight information regarding children who attended the school within the MSOA rather than specifically representing the weight of children residing in that MSOA. We also do not know how and whether children engage with their local food environment with the data available to us.

## CONCLUSION

This research finds evidence to support that planning policy could be used as part of an arsenal of tools to reduce inequalities in childhood OWOB. This policy may only be effective in areas with a high concentration of fast‐food outlets. Those who have authority over planning in communities should consider how the planning approach can be adjusted accordingly and applied within their area to promote healthy weight in their communities.

## FUNDING INFORMATION

This study is funded by the National Institute for Health and Social Care Research (NIHR; Applied Research Collaboration North East and North Cumbria [NIHR200173]). The views expressed are those of the author(s) and not necessarily those of the NIHR or the Department of Health and Social Care. Heather Brown is also supported by the NIHR Applied Research Collaboration North West Coast (NIHR200182). An earlier version of this paper is available as a working paper: Xiang et al., Does Using Planning Policy to Restrict Fast Food Outlets Reduce Inequalities in Childhood Overweight and Obesity? IZA Discussion Papers, No. 15795, Institute of Labor Economics (IZA), 2022, https://www.econstor.eu/handle/10419/272422

## CONFLICT OF INTEREST STATEMENT

The authors declared no conflict of interest.

## Supporting information


**APPENDIX:** Supplementary Information.
