# Supplementary material for: Planning policies to restrict fast food and inequalities in child weight in England: a quasi‐experimental analysis
Source: Obesity (Silver Spring). 2024 Oct 23;32(12):2345–53. doi: 10.1002/oby.24127 (PMC11589531; doi:10.1002/oby.24127)
Supplement: Supplementary file 1 — APPENDIX: Supplementary Information. [file OBY-32-2345-s001.docx]

**Appendix**

**Box A1:** *Types of Planning Guidance*

| Types of Planning Guidance |
| --- |
| 1. School exclusion zone (restricting planning permission for new fast-food outlets usually within 400 metres of a school) |
| 1. Limiting the density of fast-food outlets (planning permission for new fast-food outlets will be rejected if a certain threshold number of fast-food outlets has been reached) |
| 1. Restricting new fast-food outlets based upon local childhood obesity rates (restricting planning permission for new fast-food outlets in areas where more than a certain threshold percentage of children are living with obesity) |

| **Table A1. Count of Children in each MSOA in Gateshead by year** | | | | | | | | | | |
| --- | --- | --- | --- | --- | --- | --- | --- | --- | --- | --- |
|  | **2011** | **2012** | **2013** | **2014** | **2015** | **2016** | **2017** | **2018** | **2019** | **2020** |
| **MSOA code** | **(1)** | **(2)** | **(3)** | **(4)** | **(5)** | **(6)** | **(7)** | **(8)** | **(9)** | **(10)** |
| **E02001682** | 230 | 235 | 220 | 235 | 230 | 225 | 245 | 240 | 260 | 265 |
| **E02001683** | 250 | 250 | 245 | 230 | 220 | 240 | 240 | 265 | 245 | 270 |
| **E02001684** | 175 | 175 | 175 | 195 | 190 | 170 | 160 | 175 | 200 | 215 |
| **E02001685** | 270 | 260 | 270 | 250 | 240 | 250 | 260 | 270 | 275 | 280 |
| **E02001686** | 195 | 190 | 165 | 150 | 150 | 175 | 175 | 180 | 175 | 185 |
| **E02001688** | 180 | 165 | 160 | 165 | 170 | 205 | 220 | 215 | 210 | 220 |
| **E02001689** | 155 | 130 | 165 | 160 | 180 | 140 | 140 | 135 | 145 | 160 |
| **E02001690** | 240 | 215 | 185 | 180 | 190 | 190 | 190 | 205 | 250 | 245 |
| **E02001691** | 165 | 165 | 185 | 185 | 205 | 195 | 195 | 195 | 205 | 220 |
| **E02001692** | 230 | 225 | 235 | 245 | 235 | 235 | 230 | 230 | 230 | 255 |
| **E02001693** | 145 | 145 | 160 | 150 | 160 | 170 | 165 | 165 | 140 | 165 |
| **E02001694** | 195 | 210 | 190 | 185 | 170 | 170 | 175 | 200 | 190 | 185 |
| **E02001695** | 190 | 190 | 170 | 190 | 190 | 190 | 180 | 185 | 180 | 170 |
| **E02001696** | 320 | 295 | 310 | 310 | 315 | 310 | 330 | 350 | 320 | 280 |
| **E02001697** | 180 | 195 | 230 | 230 | 230 | 230 | 250 | 225 | 205 | 200 |
| **E02001698** | 200 | 185 | 190 | 175 | 185 | 185 | 185 | 180 | 185 | 195 |
| **E02001699** | 205 | 215 | 205 | 200 | 195 | 180 | 185 | 175 | 180 | 180 |
| **E02001700** | 300 | 310 | 295 | 315 | 325 | 360 | 345 | 310 | 330 | 335 |
| **E02001701** | 105 | 115 | 160 | 175 | 155 | 145 | 135 | 150 | 160 | 185 |
| **E02001702** | 275 | 245 | 275 | 270 | 290 | 295 | 315 | 320 | 315 | 315 |
| **E02001703** | 190 | 185 | 155 | 165 | 145 | 165 | 150 | 155 | 150 | 165 |
| **E02001704** | 260 | 225 | 205 | 180 | 190 | 205 | 200 | 195 | 195 | 195 |
| **E02001705** | 185 | 180 | 170 | 170 | 170 | 190 | 205 | 215 | 225 | 235 |
| **E02001706** | 180 | 180 | 205 | 205 | 225 | 215 | 230 | 235 | 250 | 260 |
| **E02001707** | 250 | 240 | 225 | 230 | 235 | 250 | 275 | 290 | 290 | 270 |
| **E02006841** | 100 | 110 | 115 | 115 | 95 | 95 | 100 | 120 | 120 | 130 |
| **E02006842** | 95 | 100 | 120 | 125 | 115 | 115 | 125 | 130 | 130 | 120 |

**Appendix A2: Sensitivity Analysis**

**Sensitivity analysis: The dynamic treatment effects**

One of the key assumptions of DID model is the `parallel trends assumption’ between the treatment and control groups. To address this concern, we estimated the dynamic effects of policy intervention using the model as shown in Equation (2).

${OWOB}_{it}=\beta_{1}{Treat}_{i}\times{Pre}_{t}^{-4}+\beta_{2}{Treat}_{i}\times{Pre}_{t}^{-3}+\beta_{3}{Treat}_{i}\times{Pre}_{t}^{-2}+\beta_{4}{Treat}_{i}\times{Post}_{t}^{-1}+\beta_{5}{Treat}_{i}\times{Post}_{t}^{+1}+\beta_{6}{Treat}_{i}\times{Post}_{t}^{+2}+\beta_{7}{Treat}_{i}\times{Post}_{t}^{+3}+\beta_{8}{Treat}_{i}\times{Post}_{t}^{+4}+\beta_{9}{Treat}_{i}\times{Post}_{t}^{+5}+\alpha_{i}+\tau_{t}+\varepsilon_{it}$ (2)

Where, ${Pre}_{t}^{-4}$, ${Pre}_{t}^{-3}$, ..., ${Post}_{t}^{+4}$, and ${Post}_{t}^{+5}$, are the year dummy variables for 2011, 2012, …, 2019, and 2020 respectively. The interaction term for the intervention year 2015, Treat_i_*Pre_t_^0^, is the base case, and therefore is omitted from the equation. α_i_ is the MSOA dummy variable. τ_t_ is the time dummy variable. We are interested in β_1_, β_2_, β_3_, and β_4_. If our setting did not violate the parallel trend assumption, β_1_, β_2_, β_3_, and β_4_ should not be statistically significantly different from 0.

An alternative parallel trend test is conducted as an additional robust test for the parallel trends assumption. This test used the Stata command `estat ptrends’. This test assumes that there was a linear trend in both treatment and control groups and then estimates pre-intervention slope differences between the two groups.

**Sensitivity analysis: Placebo tests**

As an additional sensitivity analysis, we used the pre-intervention data from 2011 to 2015. Three placebo treatment periods (2012, 2013, and 2014) are introduced. Then, we estimated a DID model to examine the `effects’ of the placebo interventions on year 6 OWOB. We would expect to see no significant association.

**Sensitivity analysis: Alternative propensity score matching approach**

There is an alternative way to define the density of fast-food outlets. This may lead to a different selection of the control groups and therefore lead to a different estimate of the treatment effects. The alternative definition is the number of fast-food outlets per km^2^. Following the same PSM method, a different set of control groups is identified. Then, the new control groups are used to examine if our main results are sensitive to the alternative control groups.

**Appendix A3: Sensitivity Analysis Results**

**The dynamic treatment effects**

Table A2 presents results estimating the dynamic effects of planning policy as well as examining the parallel trend assumption. We found that, for the overall estimates at the MSOA level, some of the pre-intervention interaction terms (i.e., Treat * Pre^-4^ – Treat * Pre^-1^) are statistically significant suggesting violation of the parallel trend assumption. Looking at the analysis by area level deprivation, the pre-intervention interaction terms are statistically insignificant for the 2^nd^ and 5^th^ deprivation quintiles, which means the parallel trends hold in these two quintiles. However, this is not the case in the 1^st^, 3^rd^, and 4^th^ deprivation quintiles. An alternative parallel trend tests (estat ptrends) finds similar results (see Appendix Table A3).

**Sensitivity analysis: Placebo tests**

In Table A4, we report results from the pre-treatment placebo tests. We proposed three placebo interventions in 2012, 2013, and 2014, respectively. The placebo interventions should have no statistically significant impacts on the prevalence of year 6 OWOB as the actual intervention was adopted after 2015. We found no statistically significant association in the second and the fifth IMD quintiles. Results from the fourth IMD quintile show a weakly significant placebo impact. Some of the placebo interventions in IMD quintile 1 and 3 have statistically significant ‘impacts’ on the prevalence of year 6 OWOB. This suggests that our results from IMD quintile 2 and 5 are robust to the placebo tests, in keeping with our findings from the sensitivity in analysis in Tables A2 and A3.

**Sensitivity analysis: Alternative propensity score matching approach**

In Table A5, we present estimates from the alternative control group. The overall results and results from IMD quintiles 1, 2, 3, and 5 are similar to our estimates presented in Table 3 and 4. Results from the IMD quintile 4 is sensitive to the alternative PSM approach.

**Sensitivity Analysis: Sub-sample analysis**

Our sensitivity analyses suggest some violations of the parallel trend assumption for the analysis at MSOA level and sub-group analysis for IMD quintiles 1, 3, and 4. Results from Table A2 imply the violation of parallel trend assumption mostly stems from data in 2011 and 2012. To address this potential concern, we re-estimated the DID model using data from 2013 to 2020. Results are reported in Table A6, which are similar with our mains results. The only difference is that the treatment effects on the 3^rd^ IMD quintile are not statistically significant in Table A6 whereas they are in Table 4.

| **Table A2. The dynamic effects of planning policy (Test of Parallel Trends Assumption)** | | | | | | |
| --- | --- | --- | --- | --- | --- | --- |
|  | **Overall** | **Q1** | **Q2** | **Q3** | **Q4** | **Q5** |
|  | **(1)** | **(2)** | **(3)** | **(4)** | **(5)** | **(6)** |
| Treat * Pre^-4^ (2011) | 2.446^**^ | 6.933 | 3.340 | 4.998^**^ | 5.978^**^ | -1.909 |
|  | (0.958) | (3.797) | (2.019) | (2.173) | (2.491) | (2.614) |
| Treat * Pre^-3^ (2012) | 2.316^**^ | 7.523^**^ | 3.996 | 7.174^***^ | 4.040 | -3.148 |
|  | (0.948) | (3.136) | (3.634) | (1.265) | (2.528) | (3.040) |
| Treat * Pre^-2^ (2013) | 1.216 | 5.333^*^ | 1.581 | 5.810^**^ | 3.337 | -2.632 |
|  | (0.893) | (2.723) | (2.100) | (2.410) | (2.288) | (2.399) |
| Treat * Pre^-1^ (2014) | 0.657 | 1.514 | 0.482 | 1.371 | 0.687 | -0.085 |
|  | (0.802) | (1.772) | (2.213) | (1.540) | (1.901) | (1.772) |
| Treat * Post^+1^ (2016) | -0.346 | 0.815 | -1.202 | -0.899 | 2.687^**^ | 0.445 |
|  | (1.143) | (1.286) | (0.882) | (3.265) | (1.211) | (1.367) |
| Treat * Post^+2^ (2017) | 0.001 | 3.887 | -1.232 | -1.538 | 4.822^**^ | 0.362 |
|  | (1.169) | (3.586) | (1.487) | (2.510) | (1.750) | (3.208) |
| Treat * Post^+3^ (2018) | 0.862 | 6.583 | -0.879 | -1.462 | 5.396^*^ | 0.735 |
|  | (1.204) | (4.392) | (1.873) | (2.401) | (2.655) | (2.454) |
| Treat * Post^+4^ (2019) | 0.531 | 9.142^*^ | -5.553^***^ | 0.940 | 3.949 | -0.224 |
|  | (1.255) | (4.876) | (1.673) | (2.076) | (2.489) | (3.693) |
| Treat * Post^+5^ (2020) | 0.782 | 6.715 | -5.679^**^ | 1.785 | 6.245^**^ | -2.488 |
|  | (1.229) | (4.871) | (2.506) | (2.558) | (2.088) | (4.695) |
|  |  |  |  |  |  |  |
| Year Dummy | Yes | Yes | Yes | Yes | Yes | Yes |
| MSOA Dummy | Yes | Yes | Yes | Yes | Yes | Yes |
|  |  |  |  |  |  |  |
| N (Number of MSOAs×Years) | 540 | 100 | 120 | 100 | 120 | 100 |
| R-squared | 0.448 | 0.264 | 0.377 | 0.267 | 0.192 | 0.223 |

| *Note:* Column (1) contains a dummy for IMD quintile. Q1-5 refer to the IMD quintile 1-5 respectively. Constants are included but not reported. Robust standard errors are shown in parentheses. N is the number of MSOAs×Years. * p < 0.10, ** p < 0.05, *** p < 0.01  Table A3 presents the results for the parallel trend test with a Stata command `estat ptrends’. Specifically, `estat ptrends’ employs the following model to test the parallel trend assumption,  ${OWOB}_{idt}=\alpha+\beta{Treat}_{id}*{Post}_{td}+t\theta_{1}{Treat}_{id}{*Pre}_{td}+t\theta_{2}{Treat}_{id}*{Post}_{td}+\varepsilon_{idt}$  Where, the subscript d indicates a deprivation quintile. Pre is a pre-intervention time indicator that is equal to 1 if the year is before or in 2015 and 0 otherwise. Post is a post-treatment indicator that is set to 1 if the year is after 2015 and 0 otherwise. Thus, θ_1_ is the parameter of coefficient that estimates the pre-intervention trend difference between the treatment and control groups. The null hypothesis of `estat ptrends’ test is that there are parallel trends before the intervention adopted (i.e. θ_1_ = 0). A F-test was used to test this hypothesis.   \| **Table A3. Alternative parallel-trends test** \| \| \| \| --- \| --- \| --- \| \|  \| F-statistic \| P-value \| \|  \| (1) \| (2) \| \| IMD Quintile 1 \| 3.83 \| 0.08 \| \| IMD Quintile 2 \| 2.80 \| 0.12 \| \| IMD Quintile 3 \| 7.19 \| 0.03 \| \| IMD Quintile 4 \| 5.92 \| 0.03 \| \| IMD Quintile 5 \| 0.86 \| 0.38 \| \| *Note*: This table reports results from the parallel trend test with a Stata command `estat ptrends’ by IMD quintile. The null hypothesis is that the parallel trends exist between the treatment and control groups before the intervention. A F-test was used to test the null hypothesis. \| \| \| |
| --- | --- | --- | --- | --- | --- | --- | --- | --- | --- | --- | --- | --- | --- | --- | --- | --- | --- | --- | --- | --- | --- | --- | --- | --- | --- | --- | --- |

| **Table A4. Pre-treatment Placebo tests (Test of parallel trends assumption)** | | | | | | |
| --- | --- | --- | --- | --- | --- | --- |
|  | **Overall** | **Q1** | **Q2** | **Q3** | **Q4** | **Q5** |
|  | **(1)** | **(2)** | **(3)** | **(4)** | **(5)** | **(6)** |
| **Placebo Year 2012** | -2.776^**^ | -4.945^**^ | 3.340 | -3.692 | -3.668^*^ | 1.623 |
|  | (1.228) | (1.964) | (2.019) | (2.207) | (2.106) | (2.403) |
| **Placebo Year 2013** | -3.118^**^ | -5.839^***^ | 3.996 | -5.309^**^ | -4.108^*^ | 2.521 |
|  | (1.267) | (2.087) | (3.634) | (2.217) | (2.076) | (2.426) |
| **Placebo Year 2014** | -2.825^*^ | -5.326^*^ | 1.581 | -4.838^**^ | -3.511 | 1.943 |
|  | (1.619) | (2.874) | (2.100) | (2.268) | (2.711) | (3.395) |
|  |  |  |  |  |  |  |
| N (Number of MSOAs×Years) | 270 | 50 | 60 | 50 | 60 | 50 |
| *Note:* This table reports results from placebo tests by IMD quintile. 2012, 2013, and 2014 are the proposed timing for three different placebo treatments. Constants, Gateshead dummies, and post-intervention dummies are included but not reported. Robust standard errors are shown in parentheses. N is the number of MSOAs×Years. * p < 0.10, ** p < 0.05, *** p < 0.01 | | | | | | |

| **Table A5. Alternative matched sample using number of fast-food outlets per km^2^** | | | | | | |
| --- | --- | --- | --- | --- | --- | --- |
|  | **Overall** | **Q1** | **Q2** | **Q3** | **Q4** | **Q5** |
|  | **(1)** | **(2)** | **(3)** | **(4)** | **(5)** | **(6)** |
| Treat | 2.331^***^ | 2.427^**^ | 3.706^***^ | 1.436 | 3.219^***^ | -0.066 |
|  | (0.439) | (0.934) | (0.759) | (1.065) | (0.937) | (1.221) |
| Post | 1.439^***^ | 0.960 | 3.822^***^ | 4.609^***^ | -1.994^*^ | -0.347 |
|  | (0.482) | (0.717) | (0.686) | (1.059) | (1.085) | (1.293) |
| Treat * Post | -1.072 | 1.986 | -4.255^***^ | -6.490^***^ | 3.640^**^ | -0.418 |
|  | (0.703) | (1.511) | (1.155) | (1.413) | (1.396) | (1.918) |
|  |  |  |  |  |  |  |
| N (Number of MSOAs×Years) | 520 | 100 | 120 | 100 | 120 | 80 |
| R-squared | 0.484 | 0.231 | 0.204 | 0.246 | 0.337 | 0.006 |
| *Note:* Column (1) contains a dummy for IMD quintile. Q1-5 refer to the IMD quintile 1-5 respectively. There are 80 number of observations in IMD quintile 5 because one Gateshead MSOA was not able to be matched with any MSOA in the other local authorities. Constants are included but not reported. Robust standard errors are shown in parentheses. N is the number of MSOAs×Years. * p < 0.10, ** p < 0.05, *** p < 0.01 | | | | | | |

| **Table A6. Sub-sample (from 2013 to 2020) analysis** | | | | | | |
| --- | --- | --- | --- | --- | --- | --- |
|  | **Overall** | **Q1** | **Q2** | **Q3** | **Q4** | **Q5** |
|  | **(1)** | **(2)** | **(3)** | **(4)** | **(5)** | **(6)** |
| Treat | 0.865 | 0.690 | 2.345^**^ | 0.509 | -0.537 | 1.302 |
|  | (0.599) | (1.334) | (1.086) | (1.607) | (1.272) | (1.410) |
| Post | 0.957 | 1.019 | 3.114^***^ | 1.788 | -1.018 | -0.156 |
|  | (0.643) | (1.267) | (0.953) | (1.580) | (1.461) | (1.858) |
| Treat * Post | 0.149 | 3.146 | -3.597^***^ | -2.629 | 3.278^*^ | 0.672 |
|  | (0.821) | (1.915) | (1.365) | (1.894) | (1.751) | (2.204) |
|  |  |  |  |  |  |  |
| N (Number of MSOAs×Years) | 432 | 80 | 96 | 80 | 96 | 80 |
| R-squared | 0.472 | 0.173 | 0.105 | 0.051 | 0.066 | 0.028 |
| *Note:* Column (1) contains a dummy for IMD quintile. Q1-5 refer to the IMD quintile 1-5 respectively. Constants are included but not reported. Robust standard errors are shown in parentheses. N is the number of MSOAs×Years. * p < 0.10, ** p < 0.05, *** p < 0.01 | | | | | | |
